# Advances in brain barriers and brain fluids research in 2021: great progress in a time of adversity

**DOI:** 10.1186/s12987-022-00343-x

**Published:** 2022-06-09

**Authors:** Richard F. Keep, Hazel C. Jones, Lester R. Drewes

**Affiliations:** 1 Department of Neurosurgery, University of Michigan, R5018 BSRB, 109 Zina Pitcher Place, Ann Arbor, MI 48109-2200 USA; 2 Bicester, UK; 3 Department of Biomedical Sciences, University of Minnesota Medical School Duluth, Duluth, MN 55812 USA

## Abstract

This editorial highlights advances in brain barrier and brain fluid research in 2021. It covers research on components of the blood–brain barrier, neurovascular unit and brain fluid systems; how brain barriers and brain fluid systems are impacted by neurological disorders and their role in disease progression; and advances in strategies for treating such disorders.

## Introduction

Despite the stresses induced by the COVID-19 pandemic, 2021 was an excellent year for publications related to blood–brain barriers and fluids of the central nervous system. In this editorial, the Editors-in-Chief of *Fluids and Barriers of the CNS* highlight some of those studies and underlying areas of research. As always, such a review cannot cover all of the excellent studies that were published last year. Our apologies for publications that are omitted, our aim is to direct readers towards areas of research to facilitate further exploration.

## Elements of the blood–brain barrier, neurovascular unit and brain fluid systems

### Brain endothelial cells

Brain endothelial tight junctions (TJ) are an essential component of the blood-brain barrier/neurovascular unit (BBB/NVU). The TJs are formed of multiple transmembrane (e.g., claudin-5 and occludin) and cytoplasmic plaque proteins which are also linked to the cell cytoskeleton. The junctions where three endothelial cells meet express the proteins tricellulin and angulin-1. The work of Castro Dias et al. [1] indicate that these tricellular junctions are important sites of T cell diapedesis suggesting a potential target to regulate neuroinflammation.

There can be important crosstalk between TJ proteins. For example, Winkler et al. [2] have examined the impact of the loss of claudin-3 on other TJ proteins and the response of the BBB to stroke. There is also crosstalk between the proteins involved in different types of brain endothelial junctions (TJ, adherens and gap junctions). Anquetil et al. [3] found that inactivating phosphoinoside 3-kinase beta protected against vascular hyperpermeability, brain edema and neuroinflammation and reduced infarct volume in a mouse model of cerebral ischemia. These effects are mediated by stabilization of cell–cell junctions through increased recycling of VE-cadherin (an adherens junction protein) to the cell membrane.

The cell cytoskeleton is important in regulating many brain endothelial functions including TJs. For example, conditional endothelial knockdown of nonmuscle myosin heavy chain IIa reduces BBB hyperpermeability and preserves TJ integrity in a mouse model of cerebral ischemia [4]. Wu et al. [5] found that neuroregulin-ErbB receptor signaling regulates the endothelial cytoskeleton, barrier permeability and angiogenesis. While most attention has focus on the role of actin and microtubules in regulating brain endothelial function, recent evidence also indicates an important role for the intermediate filament protein, vimentin [6].

Gindorf et al. [7] have recently shown that the metalloprotease meprin beta (Mep1b) is a novel regulator of brain endothelial TJs. Transfecting brain endothelial cells with Mep1b reduced claudin-5 protein expression and increased permeability, whereas a global Mep1b KO mouse had increased TJ protein expression and reduced brain endothelial permeability. Like matrix metalloproteinases, Mep1b may be an important regulator of brain endothelial TJ proteins.

A variety of efflux transporters (e.g., p-glycoprotein) expressed by brain endothelial cells are important for limiting the entry of many potential therapeutic agents into brain. Even in conditions where there is barrier ‘disruption’, such as in brain tumors, efflux transport can limit drug delivery to sites of action [8]. In addition to impacting drug delivery, efflux transport has an important role in clearing endogenous substrates from the brain. For example, p-glycoprotein is involved in clearing β-amyloid from brain. Ding et al. [9] have found that inhibiting p-glycoprotein degradation and thereby increasing its expression can enhance β-amyloid clearance from the brain in a mouse model of Alzheimer’s disease. In mice, Zhang et al. [10] have reported that efflux transporters have a circadian rhythm, with efflux being highest in the active phase (dark or night) and lowest in the resting phase (light or day). A circadian rhythm in efflux transporter activity could have important consequences in the clinic by establishing an optimal time when medications should be given in patients.

Brain endothelial cells express the glucose transporter Glut-1 and is essential for brain growth and development in humans during the postnatal period. Haploinsufficiency in the gene encoding Glut-1 also disrupts angiogenesis and brain function. Interestingly, Tang et al. [11] found that endothelial-specific Glut-1 deletion has the greatest effect on angiogenesis and brain function early in development. Reducing Glut-1 later in development (after angiogenesis) only had a mild or little effect.

The properties of the cerebral endothelium vary as it transitions between arterioles, capillaries and venules. Kucharz et al. [12] have found that post-capillary venules are the major site for transcytosis of therapeutic nanoparticles. Low density lipoprotein receptor-related protein-1 (LRP1) is involved in receptor mediated endocytosis. Storck et al. [13] have used conditional knockdown mice to abate LRP1 in brain endothelial cells and the choroid plexus epithelium. That caused protease mediated TJ degradation, loss of barrier integrity and reduced p-glycoprotein levels. Again, this suggests an interplay between different barrier functions (such as endocytosis with TJs and carrier-mediated transport).

Retinal and brain endothelial cells are essential components of the blood-retinal barrier and the BBB/NVU, respectively. However, despite similarities in function, Li et al. [14] have described differences between these two cell types including in junction proteins, transporters and signaling pathways.

### Neurovascular unit

The NVU has essential roles in brain function including linking changes in brain activity to altered cerebral blood flow (neurovascular coupling) and regulating BBB function. Astrocytes are a critical part of the NVU. Perivascular astrocyte endfeet cover nearly the entire abluminal surface of cerebral endothelial cells and associated pericytes, and they highly express certain proteins (e.g., aquaporin-4). Stokum et al. [15] compared the proteome between astrocyte endfeet and somata and identified over 500 proteins (a fifth of all proteins detected) that were endfeet-enriched. This should provide important insights into astrocyte endfeet function. Wang et al. [16] have examined the importance of Hedgehog signaling in astrocytes using conditional inactivation. They found localized specific defects in endothelial cell function (transcytosis) in rostral hypothalamus and spinal cord but not other regions (e.g., in cortex). NVU regulation appears to differ by brain region.

The function of astrocytes is altered in brain injury and disease with the production of reactive astrocytes which have been broadly classified into A1 and A2 phenotypes. Jain et al. [17] have found that astrocyte expression of the transcription factor, serum response factor (SRF), is required to maintain astrocytes in a non-reactive state. Conditional deletion of SRF resulted in astrocytes expressing more A2 reactive astrocyte marker genes.

Pericytes have multiple functions at the NVU including barriergenesis and angiogenesis [18]. Torok et al. [19] have reported that pericyte-deficient mice die after induction of experimental autoimmune encephalomyelitis (EAE; a model of multiple sclerosis) due to a massive leukocyte infiltration. This suggests an important role of pericytes in regulating neuroinflammation. Sun et al. [20] have implicated pericyte loss in increased brain endothelial transcytosis in a model of cerebral hypoperfusion. Zhang et al. [21] have found an important role in endothelial:pericyte signaling via epoxyeicosatrienoates in functional hyperemia. A major role of the NVU is regulation of cerebral blood flow (CBF) to meet changes in brain energy demand (neurovascular coupling). There are still major controversies over the relative role of arterioles vs. capillaries (pericytes) in regulating CBF as well as the importance of different signaling pathways in regulating flow. Those controversies have been recently reviewed [22]. Neurovascular coupling may vary between different areas of the brain. Shaw et al. [23] compared hippocampus and neocortex as the former is damaged early in Alzheimer’s disease and is particularly vulnerable to hypoxia-induced injury. They found that neurovascular coupling, CBF, and hemoglobin oxygenation were all reduced in the hippocampus. These were linked to differences in endothelial and pericyte function between the two regions.

Mae et al. [24] have examined the effects of pericyte loss on the cerebral endothelial cell at the single cell level. They found that pericytes are important in establishing endothelial gene expression patterns and differences along the vascular tree (arterio-venous zonation) and in inducing angiogenic quiescence. In contrast, pericyte loss only impacted a limited set of BBB/NVU functions. Similarly, using co-cultures, Kurmann et al. have used transcryptomic analysis to examine the effects of pericytes on brain endothelial cells [25] and vice versa [26]. Pathway enrichment analyses showed particular effects on inflammatory and extracellular matrix pathways. Loss of endothelial-pericyte crosstalk has been proposed to be a major contribute to dementia pathology in Alzheimer’s disease and cerebral small vessel disease (reviewed in [27]). Crosstalk and physical interactions between brain endothelial cells and pericytes may occur at specialized ‘peg-and-socket’ structures. Ornelas et al. [28] have completed a detailed morphological investigation of these sites of interaction.

Although most attention has focused on signaling between endothelial cells and adjacent cells of the NVU (e.g., pericytes/astrocytes and other mural cells) there is also signaling along the vascular tree. Mughal et al. [29] have described how traumatic brain injury (TBI) disrupts brain endothelial Kir2.1 channels which impairs capillary-to-arteriole electrical signaling which in turn impacts brain hemodynamics.

Other components of the NVU are perivascular macrophages and microglia. Walsh et al. [30] examined the relationship between microglial activation and BBB/NVU hyperpermeability in sporadic cerebral small vessels disease. They found that while both occurred, they were separated spatially. Perivascular fibroblasts are also components of the NVU in arterioles and venules, but not the capillary bed [31]. Manberg et al. [32] reported altered activation of those cells in the pre-symptomatic stage in models of amyotrophic lateral sclerosis (ALS). In addition, increased plasma levels of SPP1, a marker for perivascular macrophages, at ALS diagnosis predicted shorter survival in those patients.

### Choroid plexus

The choroid plexuses are present in the lateral, 3rd and 4th cerebral ventricles. Although the choroid plexuses have similarities in morphology, recent data have indicated important differences. Dani et al. [33] characterized the mouse choroid plexus between ventricles and during development and aging using single-cell and single-nucleus RNA sequencing as well as spatially mapping specific proteins and mRNAs. This provides an essential resource. In mouse, Kaiser et al. [34] have identified an important role of Wnt5a in 4th ventricle choroid plexus morphogenesis and size. Vong et al. [35] found a crucial role of the transcription factor Sox9 at the 4th ventricle choroid plexus with genetic deletion causing a hyperpermeable blood-CSF barrier and upsetting CSF composition. Those effects were mimicked by reducing Col9a3 expression and collagen IX levels were required for correct epithelial polarity and TJ assembly.

One choroid plexus function is CSF secretion. Water movement across barrier tissues is traditionally hypothesized to follow osmotic gradients (standing gradient hypothesis). In a recent review, MacAulay [36] cites evidence challenging this hypothesis and positing that in leaky epithelial (such as the choroid plexus) specific membrane transporters such as the sodium potassium chloride transporter, NKCC1, are directly involved in transporting water. It should be noted that NKCC1 transport across membranes is bidirectional depending on existing ion gradients and that Xu et al. [37] have recently suggested that in early development NKCC1 acts to transport ions from CSF to choroid plexus epithelium rather than the reverse.

The choroid plexus is an important site for regulating leukocyte movement between blood and brain [38]. There are also resident inflammatory cells within the choroid plexus stroma and on the apical surface of the epithelium (epiplexus cells). A recent study examined the effects of peroxiredoxin (Prx)-2, a major component of red blood cells, to explore its potential role in post-hemorrhagic hydrocephalus [39]. Intracerebroventricular Prx-2 induced hydrocephalus and this was linked to changes in the inflammatory cells at the choroid plexus.

Choroid plexus volume can be examined in humans and animals by MRI. Fleischer et al. [40] have reported an increase in choroid plexus volume in multiple sclerosis (MS) patients and in two MS-related mouse models and propose that changes in choroid plexus size reflect neuro-inflammation. Natalizumab, an antibody targeting α4-integrin currently used in MS patients, prevented increases in choroid plexus size. Ricigliano et al. [41] have also reported an increase in choroid plexus size in MS patients on MRI.

### Ependyma

There continues research focus on the role of ependymal cilia in CSF dynamics and hydrocephalus development (reviewed by [42, 43]). Two recently described genetic mouse models have cilia abnormalities. Mice with a heterozygous deletion of CWH43 develop hydrocephalus and have reduced ependymal cilia numbers [44]. Similarly, Trip6 deletion in mice results in shorter and fewer ependymal cilia and is accompanied by development of hydrocephalus. Evidence indicates an important role of Trip6 in ciliogenesis [45].

### Cerebrospinal fluid

An understudied part of the CSF system is the Reissner fiber produced in the midbrain by the sub-commissural organ (SCO) and extends caudally through the ventricular system and the central canal. It is formed by a glycoprotein SCO-spondin that has crucial roles in CSF physiology (recently reviewed by Sepulveda et al. [46]). Yang et al. [47] have described the importance of the gene Camel in ventricular system development and found that loss of Camel function resulted in the Reissner fiber not forming during development, together with hydrocephalus and scoliosis. In contrast, increasing Camel expression caused Reissner fiber misdirection.

The relative importance of different CSF drainage pathways is a focus of much research (see Proulx [48] for a detailed review) particularly with regards to clearance of endogenously-derived neurotoxic compounds (such as β-amyloid) from brain. A potential flip side to the role of the CSF (and the glymphatic system) in clearing neurotoxic compounds is that other brain areas can be exposed to the compounds, potentially causing injury. Spalloni et al. [49] have found that CSF from frontotemporal dementia patients is neurotoxic, while Wan et al. [50] found that intracerebroventricular injection of CSF from subarachnoid hemorrhage patients can cause hydrocephalus in mice.

There continues to be enormous interest in the use of CSF proteins as biomarkers, particularly in relation to dementia [51–54]. The use of CSF biomarkers to distinguish between Alzheimer’s disease and frontotemporal lobar degeneration has been examined by Cousins et al. [55]. They found that assessing neurofilament light chain in CSF provided a more accurate diagnosis than CSF total tau. Palmqvist et al. [56] found that plasma phospho-tau in combination with APOE genotyping and brief cognitive tests predicted Alzheimer’s disease dementia with an accuracy (area under the curve = 0.91) that wasn’t improved using CSF biomarkers (phospho-tau, Abeta42/Abeta40, neurofilament light chain).

In patients with relapsing multiple sclerosis, Oechtering et al. [57] have also found that intrathecal IgM synthesis is a marker for shorter times to relapse and disease severity. While most CSF biomarker studies have focused on phosphorylated and non-phosphorylated proteins, there has been recent interest in examining CSF levels of glycosylated proteins [58] and changes in CSF lipid composition as potential biomarkers for neurodegeneration [59].

There is also interest in using liquid biopsies of CSF to diagnose brain cancers. Cell-free tumor DNA from CSF and digital PCR has been used to diagnose patients with CNS lymphomas due to a characteristic MYD88 L265P mutation [60] and diffuse gliomas by detection of diagnostic mutations [61].

Limits on CSF availability may hinder CSF metabolomic studies. Panyard et al. [62] have combined metabolome-wide and a genome-wide association (GWAS) study in patients to create a model whereby publicly available GWAS data can be used to predict CSF metabolites and examine CSF-metabolite/phenotype associations in neurological disorders. Yang et al. [63] have combined proteomic data from CSF, brain and plasma with gene expression and genetic data to identify tissue-shared and tissue-specific protein quantitative trait loci (QTLs).

### Glymphatics

Since the first description in 2012 [64], the glymphatic system has engendered much interest (and some controversy). In 2021, there were 177 articles on glymphatic/glymphatic system in MedLine (Ovid). Hablitz and Nedergaard [65] have reviewed the current status of the field from the perspective of the initial study’s senior author.

One of the main areas of interest around the glymphatic system relates to clearance of potentially neurotoxic compounds from the brain, such as β-amyloid in Alzheimer’s disease. Similarly, in Huntington’s disease, Caron et al. [66] recently found that mutant huntingtin is secreted by neurons in the brain and cleared by the glymphatic system to CSF. There are still controversies as to the mechanisms by which toxic compounds and other waste products are transported through the interstitial and perivascular spaces. Dynamic contrast-enhanced MRI has enabled diffusivity to be measured in mice and Ray et al. [67] found that convection not diffusion is the dominant mechanism, especially for large molecules.

Another area that has garnered much interest relates to changes in glymphatic and CSF flow during sleep. The importance of this for brain function and how disruptions in flow may contribute to neurological disorders is being investigated and has recently been reviewed [68, 69]. In human subjects, using MRI, Tuura et al. [70] reported an increase in brain water diffusivity and CSF flow at night. Eide et al. [71] reported that sleep deprivation reduced the clearance of a tracer, gadobutrol, from the human brain and Targa et al. [72] found a correlation between depth of sleep and CSF levels of markers of axonal damage and neuroinflammation in patients.

There is debate about the role of an enlarged perivascular space, a component of the glymphatic system, in cerebral small vessel disease and Alzheimer’s disease. Gertje et al. [73] found that enlargement of these spaces was associated with small vessel disease, but their data did not support a role in the early stages of Alzheimer’s disease pathology. In contrast, Paradise et al. [74] found that severe perivascular space dilation was associated with increased chance of cognitive decline and dementia and Ciampa et al. [75] reported that genetic risk factors for Alzheimer’s disease are associated with enlarged perivascular spaces in the centrum semiovalae region of the brain.

Novel methodologies by which to study the glymphatic and meningeal lymphatics in patients may provide important clinical information. Wu et al. [76] have used 3-D isotropic contrast-enhanced T2-FLAIR magnetic resonance imaging to examine both para-arterial and meningeal lymphatic transport in patients with and without BBB disruption.

### Meninges

There continues to be an upsurge in interest in the functions of the meninges. Schafflick et al. [77] have used single cell profiling to compare tissue-resident leukocyte populations in brain parenchyma, subdural meninges, dura mater, choroid plexus and CSF and found location specific differences. In particular, the dura contained a large number of B cells and B cell precursors. This population appeared independent of skull and peripheral bone marrow. In a T helper (Th17) cell-induced neuroinflammation in rodents, Hartlehnert et al. [78] found that B cells with a follicular phenotype were exclusively found in the meninges with the transcription factor Bcl6 in Th17 cells regulating Th17-B cell interaction in the meninges. Brioschi et al. [79] have also examined B cells in the meninges. They propose that B cells derived from the calvaria mature in the meninges and acquire immune cell tolerance to CNS antigens. Furthermore, they posit that with age a population of B cells accumulates that react to CNS antigens, thus altering the immune privilege. For a recent review of different immune cell niches within the brain see Croese et al. [80].

The rediscovery of meningeal lymphatics has garnered much interest particularly in brain-body immune communication. Castranova et al. [81] have described meningeal lymphatics in the zebrafish, similar to that found in mammals. The ability to visualize these lymphatics in vivo may make this a useful model for studying their function. Brain lymphatic endothelial cells that do not form a lumen have been described and Jeong et al. [82] found that those cells take up monomeric β-amyloid42 and were involved in clearing it from the zebrafish brain.

Mezey et al. [83] have used immunohistochemistry to try and identify lymphatic endothelial cell in the human brain. They found lymphatic marker-positive cells often in close proximity to CD3-positive T cells in a variety of sites including the meninges, along the cranial nerves and in the perivascular spaces.

In 2018, channels through the skull that link meninges to skull bone marrow and provide a route for myeloid cell migration were described [84]. Now, Cugurra et al. [85] have described the role of these channels under normal conditions and during neuroinflammatory disease in both brain and spine. Monocytes using this route to enter brain appear to have a different phenotype from those entering via diapedesis across the brain endothelium.

### Brain gut axis

The gut microbiome and gut inflammation influence BBB function. Thus, for example, chronic colitis exacerbates neuroinflammation and cognitive impairment in mice [86] and the gut microbiome appears to play a major role in the BBB disruption that occurs in the spontaneously hypertensive stroke prone rats (SHRSP) compared to Wistar Kyoto rats [87]. Although most interest has been in how the gut impacts the BBB, there is also evidence that intestinal inflammation can impact the choroid plexus. Interestingly, Carloni et al. [88] found evidence that gut inflammation caused reduced permeability at the level of the choroid plexus endothelium thus protecting the brain from bacteria or bacteria-derived molecules.

Hiltensperger et al. [89] have labeled antigen-specific T cells in lymph nodes draining the gut and skin and examined their migration into brain in the experimental autoimmune encephalitis model of MS. Interestingly, T cells primed in the nodes draining gut and skin expressed different T-cell markers (P2rx7 vs. Cxcr6) and infiltrated different parts of the CNS (white vs. gray matter).

### Technology

There continues to be great interest in the use of human pluripotent stem cells (hPSCs) to produce brain microvascular endothelial cell (BMEC)-like cells [90]. Lu et al. [91] have expressed concerns that such cells express epithelial-like properties (see also [90]). This underscores the need for benchmarking models derived from such cells [92]. hPSCs are also being used to produce other cells of the NVU and Gastfriend et al. [93] have recently described a protocol for deriving pericyte-like cells.

A great advantage of using hPSC-derived models is the possibility to examine the effects of human disease or genetic mutations on barrier function. For example, Raut et al. [94] have examined the function of BMEC-like cells derived from hPSCs from patients with Alzheimer’s disease and mutations in presenilin (PSEN)1 and PSEN2 and found impaired barrier function in the PSEN1 cells.

Progress continues to be made on modeling the NVU using brain microfluidic chips seeded with various vascular cells and subjected to conditions reflective of different disease states. For example, Lyu et al. [95] and Wevers et al. [96] have developed NVU-on-a chip models for examining therapeutic strategies for stroke. Pediaditakis et al. [97] have devised brain-chips with fluid flow through vascular channels that model Parkinson’s disease and related synucleinopathies.

Another approach being employed is using human blood vessel organoids in combination with cerebral organoids. Ahn et al. [98] found that vascular cells from the blood vessel organoid penetrated the cerebral organoid and formed vessel-like structures expressing BBB molecular markers.

The use of optogenetics to modulate cell function has widely been used, particularly in neurons. There are now tools available to modulate pericyte and vascular smooth muscle cell function as reviewed in [99].

## Neurological disorders

The BBB, NVU, choroid plexus, meninges, ependyma and brain fluid dynamics are all impacted by different disease states. In addition, these changes may be therapeutic targets for limiting brain injury and dysfunction. This is a very large area of research, but examples of research published in 2021 include studies on several topics.

### SARS-CoV-2/COVID-19

The mechanisms underlying the neurological sequelae of SARS-CoV-2 (COVID-19) continue to be a matter intense research and debate [100–102]. Proposed mechanisms include secondary effects of peripheral disease including hypoxia, thrombosis and inflammatory responses; virus or viral protein entry into the CNS; or effects at the blood–brain interfaces including brain endothelial and pericyte injury.

After infection, SARS-CoV-2 RNA is rarely detected in brain. However, the ACE2 receptor, the main entry receptor for virus penetration, is abundantly expressed by a subset of brain pericytes [103, 104] suggesting that SARS-CoV-2 infection might disrupt pericyte homeostasis and lead to perivascular inflammation and a compromised BBB/NVU.

An alternate potential cause of neurological sequelae is entry of viral proteins (spike or other proteins) without RNA. Rhea et al. [105] have found evidence that the spike S1 protein can enter mouse brain after intravenous or intranasal administration (although the latter was at a lower rate). In human post-mortem tissues, Nuovo et al. [106] found S1 spike protein (but not viral RNA) in brain endothelium with localized neuronal reaction. They also found that full length S1 but not S2 spike protein could cause neurological effects in mice. Meinhardt et al. [107] have provided evidence for another route for SARS-CoV-2 entry (viral RNA and protein) into the brain, across the neural-mucosal interface in olfactory mucosa.

The BBB and the blood-CSF barriers (e.g., choroid plexus) may be sites of injury or relay systemic perturbations to the brain. Kirschenbaum et al. [108] have reported evidence of brain endothelitis, microthrombi and microbleeds in patients who died with SARS-CoV-2 infection. Wenzel et al. [109] have found that the main protease of SARS-CoV-2 (M^pro^) can cleave the protein NEMO on brain endothelial cells leading to cell death. Evidence regarding cerebral endotheliopathy and cerebrovascular involvement in SARS-CoV-2 infection has been reviewed by Kakarla et al. [110] and Whitmore and Lin [111]. Using transcriptome analysis of human tissue, Yang et al. [112] found perturbations in choroid plexus epithelial gene expression with COVID-19 that overlap with those found in chronic brain disorders.

### Stroke/traumatic brain injury

Specifically targeting the cerebral endothelium genetically has provided evidence of the critical role of those cells in ischemic brain injury. For example, in mice, Nitzche et al. [113] induced endothelial selective deficiency in sphingosine 1-phosphate (S1P) production and export as well as the S1P receptor and found exacerbation of brain injury, BBB hyperpermeability and perfusion deficits. In contrast targeting S1P signaling in lymphocytes had little effect.

Monitoring the levels of TJ proteins in plasma or CSF may be a way of assessing BBB impairment after stroke or other forms of brain injury. Andersson et al. [114] reported increases in both blood and CSF occludin and claudin-5 after hypoxia/ischemia in neonatal rats.

Stroke causes neuroinflammation with activation of resident immune cells as well as the infiltration of peripheral immune cells into brain. Thus, for example, Eidson et al. [115] have found that polymerase delta-interacting protein-2 (Poldip2) mediates vascular inflammation and leukocyte migration into brain after cerebral ischemia in mice. Poldip2 activates focal adhesion kinase (FAK), which upregulates vascular adhesion molecule-1 (VCAM-1) and promotes leukocyte recruitment. However, signals from brain across the BBB and the lymph system also impact the systemic immune response. Choi et al. [116] have reviewed the complex crosstalk between the different components of the immune system after stroke.

### Cerebral vascular malformations

Genetic mutations in KRIT1, CCM2 and PDCD10 have been identified that underlie cerebral cavernous malformations (CCM) type-1, -2 and -3. However, it is unclear why individual CCMs may exhibit sudden growth leading to neurological symptoms. Ren et al. [117] have found that in cells with a CCM loss of function mutation, somatic mutations in PI3KCA enhance phosphatidylinositol-3-kinase/mTOR signaling and induce lesion growth. Such growth can be blocked with the mTOR inhibitor rapamycin.

It is also unclear why CCMs only occur in the CNS as endothelial-specific inactivation of Krit1, Ccm2 or Pdcd10 is sufficient to cause lesion formation in mice. Lopez-Ramirez et al. [118] provide evidence for the involvement of astrocytes in lesion formation. They found increased nitric oxide production from CCM endothelium stabilizes hypoxia inducible factor (HIF)-1a in astrocytes. This in turn increases vascular endothelial growth factor and other HIF-1a target genes leading to lesion development.

Novel preclinical models of CCMs and brain arteriovenous malformations (AVMs) may help identify potential therapeutic targets. In mice, Park et al. [119] have used a novel adeno-associated virus that targets the cerebral endothelium to overexpress the oncogene KRAS. They describe the induction of brain AVMs and secondary neurologic consequences that were ameliorated with the mitogen-activated kinase kinase (MEK1/2) inhibitor trametinib.

### Brain edema and intracranial pressure

Intracranial pressure (ICP) is widely used to assess brain injury and guide therapy. However, Norager et al. [120] point out that the data on normal ICP are sparse and give reference values from the few available studies that are available. They also examine the impact of body position on normal ICP and lumbar CSF pressure.

### Hydrocephalus

A wide range of mutations have been associated with congenital hydrocephalus (e.g., [44, 45, 47, 121]). For example, Ito et al. [122] found that deficiency of the proteoglycan, Tsukushi (TSK), alters neurogenesis in the subventricular zone, disrupts ependymal structure, alters Wnt signaling and results hydrocephalus in mice. Multiple TSK variants in patients with hydrocephalus were identified.

In rodent congenital hydrocephalus models, hydrocephalus becomes apparent from late gestation. Requena-Jimenez et al. [123] performed a CSF proteomic analysis comparing control and hydrocephalic rats during development. They found a proteome shift between day 17 and 20 of gestation that coincided with the development of hydrocephalus.

Compared to congenital hydrocephalus, less is known about mutations contributing to idiopathic normal pressure hydrocephalus (iNPH) in adults. However, Yang et al. [44] have now identified loss-of-function mutations in the gene CWH43 in 15% of iNPH patients. Yang et al. [124] examined the mechanisms involved and found CWH43 deletions cause downregulation in the cell adhesion molecule L1CAM selectively in the ventricular and subventricular zones. It is known that mutations in L1CAM are a cause of X-linked congenital hydrocephalus [125].

As with many neurological diseases, machine learning has been used in the context of hydrocephalus. Thus, Hale et al. [126] used an artificial neural network in an attempt to predict which pediatric patients with hydrocephalus were at risk of shunt failure. The artificial neural network has reasonable specificity of 90% but a sensitivity of only 68%; i.e., it could effectively rule-in patients who would most likely undergo shunt failure but was not as good at ruling out those likely to experience shunt failure.

### Aging, neurodegeneration and dementia

Aging has a major impact on many neurological disorders. Propson et al. [127] have found increases in cerebrovascular inflammation, brain lymphocyte infiltration and BBB permeability during aging in mice. Those effects were ameliorated in mice with endothelial specific loss of the complement C3a receptor. Nyul-Toth et al. [128] have performed longitudinal studies in mice using intravital two-photon microscopy and optical coherence tomography to monitor changes in BBB permeability and brain vascularity during aging. They found that the permeability of single capillaries increased with age while capillary density decreased.

There continues to be enormous efforts to use immunotherapy for Alzheimer’s disease. Da Mesquita et al. [129] examined whether the efficacy of an immunotherapy targeting β-amyloid would be impacted by ablation or enhancement of meningeal lymphatic function. In a mouse Alzheimer’s disease model, ablation worsened while enhancement improved outcomes.

There is growing evidence of a role of the cerebrovasculature in Alzheimer’s disease and vascular dementia. Ries et al. [130] targeted neurovascular and BBB dysfunction with annexin A1 in mouse Alzheimer disease models. They found acute treatment could ameliorate BBB dysfunction and reduce β-amyloid levels in the brain by increasing clearance and degradation. Chronic treatment also improved memory deficits and synaptic density. Alvarez-Vergara et al. [131] have shown that despite the presence of angiogenic markers around amyloid-β plaques, vascularity is decreased. They found evidence of non-productive angiogenesis with disassembly of blood vessels and that microglia contribute to endothelial cell loss.

There is evidence that the blood–brain barriers have a role in other forms of neurodegeneration. For example, Gate et al. [132] recently found that migration of CD4+ T cells into brain has a role in neurodegeneration in Lewy body dementia.

### Brain tumors

In brain tumors, the BBB is modified forming the blood-tumor barrier (BTB). The permeability characteristics of the BTB are very heterogeneous, thus posing problems for tumor therapeutic targeting. As with the normal NVU, there is important cross talk between the endothelial cells of the BTB and surrounding brain tumor cells (see recent review by Steeg [133]).

Single-cell transcriptomic analyses have shown that individual gliomas have diverse populations of cancer cells. Carlson et al. [134] have now shown that there are diverse populations of endothelial cells within the tumor. There are tumor-derived endothelial cells, but these are relatively rare. Most of the endothelial cells are derived from surrounding brain, but even these have molecularly and cellularly unique subpopulations. This diversity of cell types increases the complexity of targeting the vasculature to treat gliomas.

Lara-Velazquez [135] have found that CSF from glioblastoma patients can initiate cancer stem cell invasion. The CSF upregulated expression of SERPINA3, the gene encoding alpha 1-antichymotrypsin (a serine protease inhibitor), in cancer stem cells. Knockdown of SERPINA3 prevented the effects glioblastoma CSF on cancer stem cell invasion, while alpha 1-antichymotrypsin induced invasion.

### Rare diseases

Leukodystrophies are monogenic diseases impacting white matter. There is relatively little known on changes at the BBB and NVU. Zarekiani et al. [136] found evidence of TJ rearrangement in 5 of 8 patients, and altered aquaporin-4 distribution in all patients examined.

### Psychiatric disorders

Recently, there has been considerable interest in the potential role of BBB dysfunction in neuropsychiatric disorders including in patients with schizophrenia (e.g., with 22q11.2 deletion syndrome). Crockett et al. [137] have used a combination of hPSC-derived BMEC-like cells, a mouse model of 22q11.2 deletion syndrome and human samples to show that BBB permeability is impaired in 22q11.2 deletion syndrome and associated with neuroinflammation. Aydogan Avsar et al. [138] have also found higher levels of serum claudin-5 in children with attention-deficit/hyperactivity disorder compared to age matched controls suggesting a potential change in BBB/NVU function.

## Drug delivery

As discussed in this section, a wide range of techniques and agents are currently being employed to deliver potentially therapeutic agents to the brain. Progress has been achieved in several approaches.

### Small molecule—chemical modification

One approach to enhance brain delivery is to chemically modify therapeutics to enhance BBB permeability. Thus, Rodriguez-Pascau et al. [139] have examined a new peroxisome proliferator-activated receptor (PPAR)-γ agonist, leriglitazone, that reaches five- to tenfold greater brain concentrations than the parent compound, pioglitazone, in rodents. They also found that leriglitazone was protective in multiple models related to X-linked adrenoleukodystrophy.

### Antibody clinical trials

There have been several large antibody-based clinical trials for CNS disorders. Dam et al. [140] examined the safety and efficacy of an anti-tau antibody (gosuranemab) for progressive supranuclear palsy (NCT03068468). However, the phase II trial failed to show evidence of neurologic efficacy even though unbound N-terminal tau levels in CSF decreased by 98%. Salloway et al. [141] examined the effects of gantenerumab (an antibody targeting amyloid-β fibrils) and solanezumab (targeting soluble amyloidβ) in dominantly inherited Alzheimer’s disease. Neither drug had a benefit on cognitive decline even though gantenerumab reduced amyloid plaques and CSF levels of total and phospho-tau. With many antibody trials for CNS disorders there have been questions of delivery to the brain. With both these trials there was evidence of target engagement suggesting questions regarding target selection.

### Receptor mediated transcytosis

There continues to be extensive efforts to use receptor-mediated transcytosis to enhance drug delivery into the brain parenchyma. For example, there has been a Phase 2/3 clinical trial of a brain penetrating fusion protein, pabinafusp alfa, for treating mucopolysaccharidosis II (Hunter syndrome) [142]. Pabinafusp alfa is a human recombinant idursulfase enzyme that uses transferrin receptor-mediated transcytosis to cross the BBB. The trial found reductions in CSF heparan sulfate concentrations and cognitive improvement in 21 of 28 patients.

Mutations in the gene GRN are a cause of frontotemporal dementia. The transferrin receptor has been targeted to deliver progranulin to mice lacking this gene [143]. Brain pathology and biochemistry changes in treated mice were ameliorated by this type of delivery.

### Blood–brain barrier disruption

A variety of approaches to disrupt the BBB are being used clinically or tested preclinically to deliver therapeutics to the brain. Intracarotid injection of hyperosmotic mannitol has been used clinically to induce transient disruption for many years. Burks et al. [144] found such disruption induces a transient sterile inflammatory response within the brain that resolves within 96 h. It is possible that this might be a method to enhance neuroinflammation as well as deliver therapeutics.

There has been considerable preclinical work on the use of focused ultrasound to disrupt the BBB and enhance brain drug delivery or immune therapy [145]. This work is now being translated to the clinic. Thus, there are clinical trials using MRI-guided ultrasound with intravenous microbubbles in Parkinson’s disease [146] (NCT03608553), Alzheimer’s disease [147] (NCT03671889) and gliomas [148] (NCT03322813).

An alternate approach to disrupting barrier tissues such as the BBB/NVU is targeting TJ proteins [149]. A number of methods have been developed to decrease or increase claudin-5 expression (reviewed in [150]). Tachibana et al. [151] used a monoclonal antibody against claudin-5 in a non-human primate and found it can cause BBB disruption. However, the therapeutic window was narrow with higher doses causing convulsions and brain edema as well as adverse systemic effects.

Another novel approach employs light excitation of circulating gold nanoparticles (AuNPs) [152]. Picosecond laser pulses generate biophysical effects that trigger a temporary increase in neurovascular permeability and involves diffusion through disrupted tight junctions. Small and large cargo are effectively delivered by barrier modulation that reverses in a few hours.

Linville et al. [153] have described a novel disruption method using a component of bee venom, the peptide melittin. It induces transient disruption of the cell–cell junctions in a human in vitro BBB model and in the mouse BBB in vivo.

### Direct cerebral injections

Although progress is being made in therapeutic delivery from blood to brain, direct intracerebral delivery is still being investigated, with for example, use of convection-enhanced delivery to increase the dispersion of the therapeutic. Thus, Pearson et al. [154] used convection-enhanced delivery of adeno-associated virus 2 to deliver the gene for the enzyme aromatic L-amino acid decarboxylase. This approach was used to deliver the vector to the midbrain of children with enzyme deficiency resulting in improvements in function 12–18 months after gene delivery.

## Conclusions

As can be seen from the above, major advances were made in brain barrier and brain fluid research in 2021 despite the ravages of COVID-19. We thank the readers, authors, reviewers and editorial board members of *Fluids and Barriers of the CNS* for their contributions to those advances and their support for the journal.

## Data Availability

Not applicable.
